# Serum brain-derived neurotrophic factor: Determinants and relationship with depressive symptoms in a community population of middle-aged and elderly people

**DOI:** 10.3109/15622975.2010.545187

**Published:** 2011-01-19

**Authors:** Boudewijn AA Bus, Indira Tendolkar, Barbara Franke, Jacqueline De Graaf, Martin Den Heijer, Jan K Buitelaar, Richard C. Oude Voshaar

**Affiliations:** 1 Department of Psychiatry, Radboud University Nijmegen Medical Centre, Nijmegen, The Netherlands; 2 Donders Institute for Brain, Cognition and Behaviour, Centre for Cognitive Neuroimaging Nijmegen, The Netherlands; 3 Department of Human Genetics, Radboud University Nijmegen Medical Centre, Nijmegen, The Netherlands; 4 Department of General Internal Medicine, Radboud University Nijmegen Medical Centre, Nijmegen, The Netherlands; 5 Department of Epidemiology and Biostatistics, Radboud University Nijmegen Medical Centre, Nijmegen, The Netherlands; 6 Nijmegen Mental Health Centre, Division of Old Age Psychiatry, Nijmegen, The Netherlands

**Keywords:** Depression, neurodegenerative disease, brain-derived neurotrophic factor (BDNF), age, gender

## Abstract

**Objectives:**

Brain-derived neurotrophic factor (BDNF) is involved in major depressive disorder and neurodegenerative diseases. Clinical studies, showing decreased serum BDNF levels, are difficult to interpret due to limited knowledge of potential confounders and mixed results for age and sex effects. We explored potential determinants of serum BDNF levels in a community sample of 1230 subjects.

**Methods:**

Multiple linear regression analyses with serum BDNF level as the dependent variable were conducted to explore the effect of four categories of potential BDNF determinants (sampling characteristics, sociodemographic variables, lifestyle factors and somatic diseases) and of self-reported depressive symptoms (Beck's Depression Inventory (BDI).

**Results:**

Our results show that BDNF levels decline with age in women, whereas in men levels remain stable. Moreover, after controlling for age and gender, the assays still showed lower serum BDNF levels with higher BDI sum scores. Effects remained significant after correction for two main confounders (time of sampling and smoking), suggesting that they serve as molecular trait factors independent of lifestyle factors.

**Conclusions:**

Given the age-sex interaction on serum BDNF levels and the known association between BDNF and gonadal hormones, research is warranted to delineate the effects of the latter interaction on the risk of psychiatric and neurodegenerative diseases.

## Introduction

Brain-derived neurotrophic factor (BDNF) belongs to the neurotrophin family of growth factors and regulates aspects of neural plasticity of neurons in multiple brain regions (Mossner et al. 2007). Significant concentrations of BDNF are also found in peripheral blood (Brunoni et al. 2008; Sen et al. 2008). In animals high correlations between cerebral BDNF expression and peripheral serum-BDNF levels have been shown (Karege et al. 2002b), suggesting a comparable relationship in humans. Along these lines, BDNF levels in humans have been linked to a wide variety of brain diseases, among which neuropsychiatric (Nakazato et al. 2003; Binder and Scharfman 2004; Hashimoto et al. 2004, 2006; Gratacos et al. 2007; Machhado-Vieira et al. 2007; Ikeda et al. 2008) and neurodegenerative diseases (Azoulay et al. 2005;Yasutake et al. 2006).

Two recent meta-analyses confirmed significantly lower serum BDNF levels for depressed patients relative to the concentrations found in healthy controls, with levels normalizing after treatment with antidepressants (Brunoni et al. 2008; Sen et al. 2008). The studies included in the meta-analyses were restricted to clinically depressed patients, most of whom were women. As experimental findings suggest that alterations in BDNF signalling are associated with depression-related behaviours in women only (Lee et al. 2007; Monteggia et al. 2007), generalization to men is questionable.

Furthermore, mixed results have been reported for associations between serum BDNF levels and age (Lang et al. 2004; Aydemir et al. 2007), although the only study conducted in older people found decreased BDNF levels with age (Ziegenhorn et al. 2007). Furthermore, in dementia patients, lower BDNF serum levels have been associated with a higher risk of neurodegenerative processes (Yasutake et al. 2006), which is in line with the neuroprotective effects of BDNF. The correlation between oestrogen and BDNF levels may not necessarily offer an explanation for the potential sex differences (Monteleone et al. 2007), but may also result in sex-specific correlations with age due to a post-menopausal drop in oestrogen levels (Simpkins and Singh 2008).

To be able to study age, sex and depressive symptoms as determinants of serum BDNF levels in humans, potential confounders have to be taken into account. Not only is BDNF increasingly considered a neurotrophin, it is also seen as an immunotrophin, epitheliotrophin and metabotrophin (Chaldakov et al. 2007), which would explain why, besides psychiatric and neurodegenerative diseases, somatic illnesses have also been related to BDNF. Somatic conditions that have been shown to correlate with BDNF levels mainly concern cardiovascular disease or cardiovascular risk factors (Ejiri et al. 2005; Geroldi et al. 2006; Hristova and Aloe, 2006; Suwa et al. 2006; Krabbe et al. 2007; Fujinami et al. 2008), which is not surprising given that serum levels may be affected by lifestyle factors such as chronic or excessive alcohol use, lack of physical exercise and smoking (Chan et al. 2008; Montag et al. 2008; Tang et al. 2008; Currie et al. 2009; Umene-Nakano et al. 2009). One study has further identified a diurnal variation in BDNF levels, suggesting the need to correct for time of sampling (Piccinni et al. 2008).

Taken together, there is ample evidence to suggest that BDNF is linked to a variety of molecular abnormalities underlying neuropsychiatric and neurodegenerative diseases. However, the findings to date were obtained in relatively small and mostly clinical samples and are thus of modest statistical power and limited generalizability, without large-scale studies having replicated the data in non-clinical cohorts or studied distinct factors affecting serum BDNF levels.

The present study was designed to do so: we looked for determinants of serum BDNF levels in a large community sample of middle-aged and elderly, with a particular emphasis on potential associations with age, sex and depressive symptoms.

## Methods

### Study population

The present study sample was drawn from the Nijmegen Biomedical Study (NBS), a population-based survey of people aged between 20 and 90 years. For details we refer to a previous publication (Hoogendoorn et al. 2006). A total of 2253 respondents were then invited to participate in a study of non-invasive measurements of atherosclerosis of whom 1517 gave their informed consent. They subsequently visited the hospital for measurements and blood sampling, as described in detail elsewhere (Holewijn et al. 2009). This latter group was hence eligible for participation in the present study. The only exclusion criterion was the use of antidepressant medication, as this has consistently been associated with BDNF levels and might mask the effect of other determinants (Brunoni et al. 2008; Sen et al. 2008).

In accordance with the Declaration of Helsinki the study protocol was approved by The Medical Ethics Committee of the Radboud University Nijmegen Medical Centre and informed, written consent was obtained from all participants.

### Serum BDNF

Following the baseline assessment and ranging from 9 to 44 months after having been drawn, serum samples were sent to the Department of Psychiatry and Neuropsychology in Maastricht (The Netherlands) for BDNF measurement, where they were stored at −80°C. Serum BDNF protein levels were measured within three months after their receipt using the Emax Immuno Assay system from Promega according to the manufacturer's protocol. The undiluted serum was acid treated, which in a dilution-dependent way reliably increased the detectable BDNF. Subsequently, serum samples were diluted 100 times and stored again at −80°C for BDNF assay the next day. After dilution, the BDNF levels were well within the range of the standard curve. The assay sensitivity threshold was ascertained at 1.56 ng/ml reflecting the minimum level of BDNF in the serum that could be reliably determined. In our pilot study we had found that BDNF levels of acid-treated samples with subsequent dilution the day preceding the BDNF assay did not differ from the levels obtained in samples following acid treatment only (i.e. without subsequent dilution) on the preceding day, or from those derived after acid treatment conducted the day of the BDNF measurement. The serum samples used in our pilot study were stored samples of six individuals that did not participate in the present study. The samples’ coefficients of variance ranged from 2.9 to 8.1%. To gauge the intra-assay variance for the present study, we analyzed two of our current samples on two different plates on the same day. The resultant coefficients of variance of 0.0 and 3.1% were both well below the maximum intra-assay variance of 8.8% as specified by the manufacturer. Greiner Bio-One high affinity 96-well plates were used and the resulting absorbance was read in duplicate using a Biorad Benchmark microplate reader at 450 nm. BDNF protein levels were expressed in ng/ml.

### Depressive symptoms

Depressive symptoms were assessed using the Beck Depression Inventory (BDI), a 21-item self-report questionnaire with excellent psychometric characteristics (Beck and Steer, 1984). Each item is rated on a 4-point scale, with 0 reflecting the absence of symptoms and 1–3 increasing levels of symptom severity. The BDI yields a total score ranging from 0 to 63. Individual items of the BDI can be considered to belong to a cognitive subscale or a somatic sub-scale (Beck and Steer 1987). Separate sum scores for both of these subscales were calculated. History of depression was assessed by a self-report question with a dichotomous outcome (yes/no).

### Possible determinants

Potential confounders of any associations between BDNF levels and depression were determined based on previous studies and categorized in four groups: sampling characteristics, sociodemographic variables, health indicators and chronic diseases.

Sampling characteristics were the time of blood withdrawal and the number of months the serum was stored at −80°C. Age, sex, and marital status were taken as the sociodemographic variables of interest. Health indicators were the presence of metabolic syndrome (MS), smoking status, physical activity, and alcohol use. MS (defined according to the International Diabetes Federation criteria (IDF 2009) was determined by physical examination and fasting vena puncture. Participants were diagnosed with the syndrome if they had central obesity (BMI >30 or above ethnic-specific waist circumference) and two or more of the following abnormalities: high blood pressure (> 130/85 mmHg), hyperlipidemia (triglyceride concentration >150 mg/dl [1.695 mmol/1]) or specific medication for this lipid abnormality, HDL cholesterol <40 mg/dl [1.03 mmol/1] in men and <50 mg/dl [1.29 mmol/1] in women or specific medication for this lipid abnormality, and fasting plasma glucose >100 mg/dl [5.6mmol/l] or previously diagnosed type II diabetes mellitus. Systolic and diastolic blood pressure were measured using an oscillometric sphygmomanometer (Criticon model no. 1846, Criticon Inc., Tampa, FL, USA), while waist circumference was determined at the level of the umbilicus and triglycerides (TG) and glucose concentrations established using commercially available enzymatic reagents (AEROSET® System, Abbott, Chicago, IL).

Smoking status, physical activity and alcohol use were assessed during a short interview and all operationalized as dichotomies: current smoker versus non-smoker, 0–1 h of weekly physical (sport) activity versus 2 h or more (Stampfer et al. 2000), and severe (>21 units for males and >14 units for females) versus non-severe use of alcohol, respectively.

As to chronic diseases, we screened for coronary artery disease (CAD) and other somatic conditions. CAD was defined and coded as present/absent based on a history of treated angina pectoris, myocardial infarction, a history of percutaneous transluminal coronary angioplasty or coronary artery bypass grafting. Other solitary or comorbid somatic conditions were lumped together as they have never been linked to BDNF serum levels before. All were coded as present/absent, with somatic disease being operationalized as receiving treatment for rheumatic disorder (or arthritis), COPD, liver disease, kidney disease or a history of or current treatment for Crohn's disease or ulcerative colitis. Dementia was determined by self-report and, if reported, crosschecked against the respondents’ medical records.

### Data analysis

The BDNF levels were normally distributed in our sample. Levels above three standard deviations (SDs) were trimmed to the 3-SD value, i.e. 17.8 ng/ml, which occurred in 11 (0.9%) cases.

Respondents with more than two missing item scores on the BDI were excluded from the analyses. In case of one or two missing items on the BDI, missings were replaced by the series mean rounded to the nearest full digit (necessary in 73 subjects (5.7%); in total 88 of 25.830 (£0.3%) items). Imputation by the series mean is a reliable method with up to 10% missing items (Shrive et al. 2006). Since the BDI scores showed a skewed distribution (skewness: 1.52, kurtosis: 3.01), we used the log-transformed BDI scores in all analyses (skewness: 0.32, kurtosis: −0.50).

Pearson's correlation coefficients were used for all univariate analyses. We performed multiple linear regression analyses to evaluate the independent determinants of serum-BDNF. To facilitate the interpretation of the age-sex interaction, rather than taking their absolute ages, we calculated and included the deviation from the mean, so that the participants’ ages were centred around the sample's mean age. First, we generated multivariate models within each domain by entering sampling characteristics, sociodemographic characteristics, health indicators and health-status variables in four separate models. Subsequently, the independent predictors from all domain-specific models with *P* values lower than 0.15 were fitted into a final multivariate model using an enter method. The association between serum BNDF level and depression was examined by adding current depressive symptoms as indicated by the log-transformed BDI sum score to the final model. All analyses were conducted with SPSS, version 16.0.

## Results

Of the 1517 older adults who consented to participate in the study of non-invasive measurements of atherosclerosis, 59 were excluded from our study because they were currently using antidepressants. Of the 1458 eligible subjects a further 228 (15.6%) were excluded due to missing data: non-return of the postal questionnaire containing the BDI (*n* = 175); three or more missing BDI item scores (*n* = 37); violation of the rules for a reliable MS or BDNF measurement (i.e. having smoked prior to testing (*n* = 3); non-adherence to the pretest fasting protocol (*n* = 2); non-adherence to abstinence from lipid-lowering medication (*n* = 5)); unavailability of serum-BDNF values due to technical problems (*n* = 6).

The subjects with missing data (228/1458, 15.6%) differed from the final study sample (*n* = 1230) with respect to age (62.0 vs. 61.2 years, r(df 1456)= 2.17, *P* = 0.03), somatic comorbidity (3 vs. 11%, r(df 1456) = −3.93, *P* < 0.001), level of alcohol use (severe 6 vs. 12%, r(df 1455) = −2.25, *P* = 0.012), and smoking (current smokers 26 vs. 16%, t(df 1454) = 3.46, *P* < 0.001). The serum BDNF levels obtained in the excluded participants with non-missing serum-BDNF levels, however, did not differ from those found in the study population.

The final study population (*n* = 1230) had a mean age of 61.1 years (SD = 5.9 years) with ages ranging from 50 through to 72 years. Of this group 614 participants (49.9%) were female. The BDI median score was 4 (interquartile range 2–7, range 0–29) and the mean serum BDNF 7.79 ng/ml (SD = 3.32 ng/ml). None of the participants was diagnosed with dementia. Table I gives an overview of the characteristics of all participants.

**Table I:** Population characteristics.

	*N* = 1230
BDNF (ng/ml, SD)	7.79 (3.32)
BDI sum score (median, IQR)	4 (2–7)
% with history of depression (*n*)	16.9 (208)
**Sampling**	
% sampling in the morning (*n*)	79.2 (974)
Months in storage (SD)	23.7 (9.5)
**Sociodemographics**	
% female (*n*)	49.9 (614)
Age (mean in years, SD)	61.2 (5.9)
% living alone (*n*)	21.4 (263)
**Health indicators**	
% current smoker (*n*)	16.1 (195)
% severe alcohol use (*n*)	11.8 (145)
% 2 or more hours of physical activity p/w (*n*)	36.6 (450)
**Disease**	
% metabolic syndrome (*n*)	27.8 (342)
% coronary artery disease (*n*)	6.7 (83)
% somatic co-morbidity other than CAD (*n*)	11.0 (135)

BDNF, brain-derived neurotrophic factor; SD, standard deviation; BDI, Beck Depression inventory; IQR, interquartile range; CAD, coronary artery disease.

**Figure 1:**
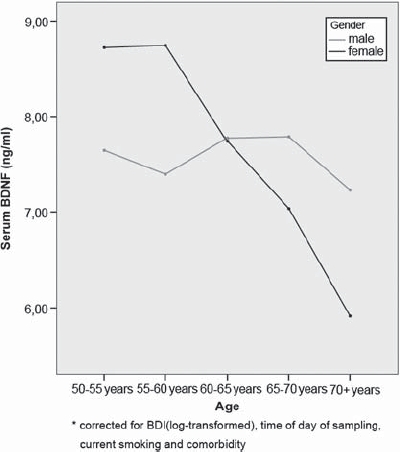
Interactice effect of age and gender on serum BDNF level.^*^

The first multiple regression models in which every domain was entered separately yielded three significant determinants. As can be seen in Table II, significantly lower BDNF levels were found when measured in the afternoon (*B* = −0.57, 95% CI −1.03 to −0.12; *t* = −2.46, *P* = 0.014), with higher age in female respondents (age-sex interaction term: *B* = −0.15, 95% CI −0.21 to 0.09; *t* = −4.73, *P* < 0.001; see also see Figure 1), and non-smokers (*B* = 0.87, 95% CI 0.37 to 1.38; *t* = 3.38,*P* = 0.001). Although the other somatic co-morbidity variable remained under the *P* = 0.15 level, it did not reach statistical significance at the *P* = 0.05 level (*B* = 0.47, 95% CI −0.76 to 1.02; *t* = 1.69, *P* = 0.091).

**Table II:** Results of the univariate and multivariate analyses per category.

	Univariate		Multivariate					
		
	*r*	*P*	*B*	95% CI of *B*	SE	β	*t*	*P*
**Model 1 (Sampling variables)**								
Time of day (0 = AM, 1 = PM)	−0.069	0.008	−0.573	−1.03–−0.12	0.233	−0.070	−2.459	0.014
Time in storage (days)	−0.014	0.31	0.000	0.00–0.00	0.000	−0.017	−0.610	0.54
**Model 2 (Demographic variables)**								
Age (years deviating from mean age)	−0.112	<0.001	0.0012	−0.03 – 0.06	0.022	0.022	0.561	0.58
Sex (0 = male, 1 = female)	0.035	0.11	0.181	−0.19 – 0.55	0.190	0.027	0.956	0.34
Interaction age X sex	n/a	n/a	−0.151	−0.21 – −0.09	0.032	−0.187	−4.734	<0.001
Marital status (1 = living alone, 2 = living together)	−0.041	0.08	−0.276	−0.73 – 0.18	0.231	−0.034	−1.199	0.23
**Model 3 (Health variables)**								
Alcohol use (male>21/week; female > 14/week)	−0.028	0.16	−0.401	−0.98 – 0.17	0.294	−0.039	−1.362	0.17
Current smoker (0 = no, 1 = yes)	0.094	<.001	0.872	0.37 − 1.38	0.258	0.097	3.379	0.001
Physical activity (0 = ≤ 1 hrs/week, 1 = >2 hrs/week)	−0.023	0.22	−0.082	−0.47 – 0.30	0.197	−0.012	−0.416	0.68
**Model 4 (Disease variables)**								
Metabolic Syndrome (IDF) (0 = no, 1 = yes)	0.007	0.39	0.035	−0.32 – 0.39	0.182	0.005	0.191	0.85
Coronary artery disease (0 = no, 1 = yes)	0.011	0.35	−0.043	−0.71 – 0.63	0.341	−0.003	−0.126	0.90
Somatic co-morbidity other than CAD (0 = no, 1 = yes)	0.044	0.05	0.473	−0.08 – 1.02	0.280	0.044	1.690	0.09

*R*, Pearson's correlation coefficient; *B*, regression coefficient; CI, confidence interval; SE, standard error of the mean; β, standardized correlation coefficient; CAD, coronary artery disease.

As Table III shows, our final model uncovered a significantly lower in BDNF levels with higher BDI sum scores (*B* = −0.56, 95% CI −1.1 to −0.02; *t* = −2.03, *P* = 0.042). Re-analysis of the age effect stratified for a low BDI (<14) and high BDI (>14) score revealed that the age effect for women was present and statistically significant in both groups (*B* = −1.25, 95% CI −1.76 to −0.75; *t* = −4.86, *P <* 0.001, respectively, *B* = −1.59, 95% CI −3.13 to −0.004; *t* = −2.07, *P* = 0.04) and also absent in both males with high BDI (>14) and a low BDI (< 14) sum scores (both *P* values > 0.61).

**Table III:** Final model with BDI scores corrected for potential confounders.

	*B*	95% CI of SE	*SE*	β	*t*	P
Constant	7.959	7.6 – 8.4	0.208		38.277	0
BDI (log-transformed)	−0.607	−1.13 – −0.09	0.265	−0.060	−2.296	2
Age (yrs)	−0.006	−0.05 – 0.03	0.020	0.011	−0.304	1
Sex (0 = male; 1 = female)	0.293	−0.04 − 0.63	0.170	.045	1.724	5
Interaction age X sex	−0.110	−0.17 – −0.05	0.028	−0.138	−3.873	0
Time of day (0 = AM; 1 = PM)	−0.405	−0.80 – −0.01	0.204	−0.050	−1.985	7
Current smoker (0 = no; 1 = yes)	0.613	0.19 – 1.06	0.215	0.072	2.843	5
Somatic co-morbidity (0 = no; 1 = yes)	0.515	−0.03 – 1.06	0.278	0.047	1.857	3

*B*, regression coefficient; CI, confidence interval; SE, standard error of the mean; β, standardized correlation coefficient.

Separate evaluation of the different subscales of the BDI, revealed that the cognitive subscale was significantly associated with serum BDNF levels (*B* = −0.544, 95% CI −1.07 to −0.016; *t* = −2.02, *P* = 0.043), whereas the somatic subscale was not (*B* = −0.312, 95% CI −1.03 to 0.406; *t* = −0.85, *P* = 0.39).

Subsequent analysis, also revealed that persons with a self-reported history of depressive illness have lower serum BDNF levels (B = −0.606, 95% CI −1.05 to −0.163; *t* = −2.68, *P* = 0.007), when corrected for potential confounders. Including a history of depressive illness and current depressive symptoms in one multivariate regression analysis led to collinearity problems. Therefore, we could not disentangle whether low serum BDNF levels reflect a state or trait characteristic.

## Discussion

To our knowledge, this is the first study of a large community sample of middle-aged and older people providing clear evidence that BDNF levels significantly decline in women with increasing age, while levels remain stable in men (see Figure 1). Moreover, and equally important, even after controlling for age and gender effects our analysis showed that BDNF levels are lower with increased levels of depressive symptoms. These effects remained significant even after correction for time of sampling and smoking, two important confounders.

### BDNF and sex and age

If the interaction is not taken into account, the sex-specific decline of BDNF levels with age may result in opposite sex effects, with higher BDNF values being found in younger women and lower BDNF values in older women relative to the levels obtained in age-matched men (Karege et al. 2002a; Laske et al. 2007). Given that the effects of sex and age were quite small, most previous studies were probably underpowered, preventing detection of significant sex differences (Laske et al. 2007). Several, mostly underpowered, studies indeed failed to find an age effect (Aydemir et al. 2007; Shimizu et al. 2003), while a small-scale study of healthy adult volunteers (mean age 41.1 years old) even reported increased BDNF levels with age (Lang et al. 2004).

The only study conducted in older adults, however, did show lower serum BDNF levels with higher age (Ziegenhorn et al. 2007), a finding we were able to replicate and extend by showing an interaction between age and sex effects. As the age effect in women was also present in subjects with a high score on BDI (> 14), this should also be taken into account in studies with a clinically depressive sample. A possible explanation for the age-sex interaction with respect to BDNF serum levels might be its relationship with gonadal hormones, as BDNF synthesis is induced by estrogen (Nagahara et al. 2009) and high correlations between plasma-BDNF and oestrogen levels have been reported in humans (Monteleone et al. 2007). Moreover, both oestrogen depletion and low serum BDNF levels have been identified as risk factors for Alzheimer's disease (Craig and Murphy 2009). Despite the cross-sectional character of the studies reviewed, the hypothesis may be put forward that a post-menopausal drop in oestrogen levels results in reduced BDNF levels, explaining the increased vulnerability of middle-aged and older women to neurodegenerative disease (Craig and Murphy 2009; Reitz et al. 2010). This hypothesis should however be tested in longitudinal studies.

### BDNF and depression

Our results extend previous studies reporting lower serum BDNF levels in patients with major depressive disorder (MDD) relative to healthy controls (Brunoni et al. 2008; Sen et al. 2008; Molendijk et al. 2010) to a broader, continuous relationship between BDNF levels and depressive symptoms in a community sample. An association between BDNF serum levels and severity of depressive symptoms has also been identified in two studies of patients with depressive disorder (Shimizu et al. 2003; Gonul et al. 2005), although one other study reported a negative result (Lang et al. 2006). Taken together, the findings suggest a linear association between the two variables that is independent of the current categorical boundaries for depression.

### BDNF and determinants

Serum BDNF levels were significantly lower if blood was drawn in the afternoon. Diurnal variations have previously been found for BDNF plasma level (Piccinni et al. 2008). However, since we only recorded whether sampling occurred before (AM) or after noon (PM), we are unable to describe the variation in BDNF levels in more detail. Of the health-behaviour variables, smoking was the only factor to show an association with BDNF levels (elevated levels in smokers). Interestingly, nicotine use has been linked to a decreased risk of dementia (Wang et al. 2009), which might point to a mediating role for BDNF. Nonetheless, as a case-control study (*n* = 40) reported reverse effects of smoking on plasma BDNF levels (Kim et al. 2007), additional research is necessary to establish whether this apparent inconsistency might be explained by altered platelet functioning in smokers, which would affect BDNF serum but not plasma levels (Nowak et al. 1987).

### Strengths and weaknesses

It remains unclear whether lower levels of circulating BDNF result from lower brain BDNF concentration. Although high correlations between serum BDNF and cerebral-BDNF levels have been shown in animal models (Karege et al. 2002b), this has, thus far, not been confirmed in humans. Despite well-established findings of lower serum BDNF in a depressed population, inferences of BDNF as a causal marker for depression are still limited. Platelets have been described as one of the many sources of BDNF: the factor is released by platelets during the clotting process resulting in a 200-fold increase in BDNF serum relative to the levels found in plasma (Rosenfeld et al. 1995). As BDNF in platelets does not originate from megakaryocytes or other precursor cells of the mature platelet (Fujimura et al. 2002), it is likely that most of the BDNF in human platelets is sequestered from blood (Nakahashi et al. 2000). Differences in platelet functioning as described in depressed patients (Ziegelstein et al. 2009), therefore, may affect BDNF independently of cerebral BDNF expression.

Due to collinearity problems between the actual level of depressive symptoms and a history of depressive illness, we weren't able to distinguish between a state or a trait effect. This may in part be explained by recall bias in assessing the history of depressive illness in our sample (as depressive symptoms may have primed persons to recall a history of depressive illness or to consider previous depressive symptoms as a depressive disorder). Future longitudinal or prospective research should be carried out to disentangle whether lower BDNF-levels reflect a state or trait characteristic.

Although we had to exclude a small proportion of our study sample,. Moreover, our comparison of the excluded participants and study population did not show a selection bias on the relevant parameters.

Although we had a large sample size, one could argue that our study be underpowered to reveal small effects, given our sample size (*n* = 1230), the largest number of predictors in one analysis (*n* = 6, see results), a power of 80% and α two-sided α of 0.05, the smallest detectable β was calculated to be 0.08. As, by convention, a β of 0.1 is considered a small effect, our study has sufficient statistical power to detect small effects.

In terms of generalizability it could be considered a limitation that our sample consisted of subjects in the age range 50–72 years. Replication in samples with a broader or other age range should be carried out. Moreover, for a proper interpretation of our results it needs mentioning that the cross-sectional design of our study does not allow causal inferences. To date, no prospective studies are available in this area of research.

Rather than a clinical population, we charted depressive symptoms in a non-hospitalized sample of middle-aged and older adults. Although there may be some bias towards the healthier individuals in the sample, we were still able to show a significant correlation between serum BDNF and depressive symptoms in this community population.

## Conclusion

Our results underscore that serum BDNF levels in humans are determined by smoking status, diurnal variation, age, sex and depressive symptoms, but that duration of storage (if at −80°C) has no impact. Future studies on BDNF in (neuro)psychiatric disorders should control for these characteristics. Moreover, the interaction between age and sex might have important implications as it may point to a clinical effect of gonadal hormones on neurotrophic growth factors. For example, BDNF might be implicated in the increased vulnerability to dementia in post-menopausal women. Here also, further corrobora-tion of the interplay of gonadal hormones and BDNF on psychiatric illnesses and neurodegenerative diseases is warranted.

## Statement of Interest

The BDNF measurements were financed by a Clinical Fellowship from the NWO (Netherlands Organisation for Scientific Research; project number 90700231) awarded to Richard C. Oude Voshaar, MD, PhD. None of the authors has any conflict of interest to declare.
